# Scoliogeny of adolescent idiopathic scoliosis: inviting contributions for a discussion based on evidence and theoretical interpretations aiming ultimately to prevention or aetiological treatment

**DOI:** 10.1186/1748-7161-8-8

**Published:** 2013-05-10

**Authors:** R Geoffrey Burwell, Peter H Dangerfield, Theodoros B Grivas

**Affiliations:** 1 Centre for Spinal Studies and Surgery, Nottingham University Hospitals Trust, Queen’s Medical Centre Campus, Derby Road, Nottingham NG7 2UH, UK; 2 University of Liverpool, Ashton Street, Liverpool L69 3GE, UK; 3 Staffordshire University, Leek Road, Stoke-on-Trent ST4 2DF, UK; 4 Royal Liverpool Children’s Hospital, Eaton Road, Liverpool L12 2AP, UK; 5 Department of Trauma and Orthopedics, "Tzanio" General Hospital, Tzani and Afendouli 1 st, Piraeus 18536, Greece

## 

This Editorial initiates a Thematic Series on the aetiology, pathogenesis and pathomechanisms (scoliogeny) of adolescent idiopathic scoliosis (AIS). It introduces the Series and invites contributions from researchers in the field and from interested parties. The aim is to develop a wide-ranging online discussion based on evidence and theoretical interpretations, In doing this, it will extend the role of the electronic focus group of the International Federated Body on Scoliosis Etiology (IBSE) which discussed nine selected topics from 2003 to 2013 [1,2].

While the relationship between scoliosis and growth was recognized forty years ago [3], progress in understanding the causation of AIS was fettered by a relative paucity of basic biological and biomechanical scientific data. Since then and subsequent to emphasis in pathogenesis on sagittal spinal plane [4] and relative anterior spinal overgrowth [5], advances in biological knowledge have enabled scoliogenic understanding to progress particularly after the year 2000 [6-8]. Knowledge limtation still restricts scoliogenic understanding for example in relation to the biochemistry and endocrinology of AIS [9] and the abnormal extra-spinal skeletal length asymmetries of AIS girls which are of unknown pathogenetic significance [2,10,11]. Research on the genetic variant hypothesis of disease for AIS has led to significant achievements [12]. Within the research forum of the British Scoliosis Research Foundation [13] there is a separate, private forum for the International Scoliosis Genetics Interest Group (ISGIG), a specialist group formed at the 2011 International Phillip Zorab Symposium. A possible role for environmental and epigenetic factors in AIS scoliogeny is uncertain [14]. The application of 3-D biomechanical methods to the trunk [15], simulation by finite element analysis [16], and biomechanical evaluation of the vicious circle hypothesis [17] are all important developments. While a neurobiological [10] and neuromuscular [18] basis for AIS scoliogeny is supported by increasing evidence, systemic metabolic abnormalities have been revealed [19-21] suggesting whole organism involvement [22] and leading to a blood test for idiopathic scoliosis [23].

Relatively less explored in scoliogenic research is how the unique combination of human form, function, size, shape, growth patterns and laterality acquired during evolution distinguishing it from other species of primate may predispose humans, but not other primates, to AIS. There is a clear need for this research as evolutionary science can be viewed as the fundamental ‘organizing principle’ of all biology [24]. Some human physical modes and the underlying genetic and physiological controls that may predisose to AIS include, upright posture with bipedalism, spinal rotations and counter-rotations during gait [25], asymmetry of the normal spine [26], ribcage [1,27,28], upper arms [11], and handedness [29,30], prolonged period of the human growth curve and adolescent spurt [31], neuro-osseous timing of growth and maturation [8,32,33], and the breaking of bilateral symmetry [34]. In experimental animals, quadrupedal or rendered bipedal [35], scoliogeny is widely studied, but the question often remains, how much applies to humans?

This Series begins with a summary of presentations during 2012 selected from two international scoliosis meetings, the International Research Society of Spinal Deformities in Poznan, Poland, and the Scoliosis Research Society in Chicago, USA [36]. This summary of the presentations reveals the diversity of current scoliogenic research, theory, and the lack of a unifying hypothesis. The latter may result from AIS being not one disease but final common pathways expressed in the trunk resulting from abnormalities in different causal paths which may interact and with mechanical factors.

Where does this leave the treatment of AIS? At present, such scoliogenic research does not add to the weaponry of treatment which limits its appeal to the pragmatic mind. There is hope that novel non-surgical preventive treatments other than braces [37] may be discovered, targeting the appropriate aetiology, and/or aetiopathogenetic pathways, to avoid surgery and maintain spinal mobility. Prevention of curve progression, if not curve initiation, by early diagnosis and treatment is the aim of research into the scoliogeny of AIS.

## Competing interests

None of the authors has any competing interests in relation to the concepts discussed in this article.

## Authors’ contributions

The idea for a Thematic Series on the aetiology of AIS was initiated by TG. The text of this Editorial was written largely by GB after discussion with PD and TG. The title was formulated by TG. All authors read and approved the final manuscript.
